# United States Veterinary Medical Association

**Published:** 1890-03

**Authors:** 


### U. S. VETERINARY MEDICAL ASSOCIATION.

AT a meeting of the Comitia Minora of the United States Veterinary Medical Association, held in New York on Saturday, Feb. 15th, it was decided that the annual meeting for 1890, which convenes the third Tuesday of September (the 16th) will be held in Chicago, Ill.

The President, Dr. Michener, appointed Dr. Huidekoper, Philadelphia, Dr. Wray, Baltimore, and Dr. Hoskins, Secretary of the Association, 12 South 37th St., Philadelphia, committee of arrangements.

This is a wise move on the part of the Association. Boston and New York have had the lion's share of the meetings for the last twenty-six years, and a few men have had to carry the burden of keeping the association in an active condition, frequently impeded by factional jealousies and lethargy on the part of many of the members in the Eastern States which is almost Oriental. Members in the West have kept up their connection with the Association, but can be readily excused for not having been often tempted on a long journey for a one day's meeting.

Now, numerous practitioners are being heard of in the West, state societies have been formed on both borders of the Mississippi, and it seems a fitting time that the parent Association should look after her offspring, and insure the harmony among all of its members which is necessary for the future welfare of the veterinary profession in this country. We are assured that every care will be taken to insure a profitable and interesting meeting. Every old member from the East should prepare to make the meeting—*September 16th*—part of his Summer's holiday, and go well armed to show the superiority of this conservative community, whilst every one from the West will undoubtedly be ready to demonstrate the advantage of his wider fields and more vast agricultural surroundings and claim his part in the National Association.

